# The complete chloroplast genome sequences and phylogenetic analysis of *Crotalaria pallida* (Leguminosae)

**DOI:** 10.1080/23802359.2021.1875926

**Published:** 2021-03-24

**Authors:** Nong Zhou, Hai-Ling Li, You Zhou, Dong-Qin Guo

**Affiliations:** a College of Biology and Food Engineering, Chongqing Three Gorges University, Chongqing, PR China; b Engineering Laboratory of Chongqing for Green Planting and Deep Processing of Genuine Medicinal Materials in Three Gorges Reservoir Area, Chongqing Three Gorges University, Chongqing, PR China

**Keywords:** *Crotalaria pallida*, complete chloroplast genome, Illumina sequencing, phylogeny

## Abstract

The complete chloroplast genome of *Crotalaria pallida* was obtained using the high-throughput sequencing technology in this article. The complete chloroplast genomes of this species were 152,658 bp in length, consisting of a large single-copy region (LSC) of 83,652 bp and a small single-copy region (SSC) of 18,028 bp, which were separated by a pair of inverted repeat (IRs) regions of 25,489 bp. The chloroplast genome contained 111 unique genes, including 77 protein-coding genes, 30 *tRNA* genes, and four *rRNA* genes. The phylogenomic relationship analysis suggested that *C. pallida* was closely related to *Lupinus* in the family of Leguminosae.

*Crotalaria pallida*, also named ‘Zhu-Shi-Dou’, is a species of genus *Crotalaria,* family Leguminosae, which is widely distributed in the tropical and subtropical regions of China, India as well as Africa and America countries (Editorial Committee of Flora of China 1998). *Crotalaria pallida* is used as folk medicine to treat scrofula, mastitis, and dysentery with stems and leaves, and to treat neurasthenia, dizziness, leucorrhea, and cancers with seeds in China (Lian 1986; Hu et al. 2017). Researches about bioactive constituents in *C. pallida* have been carried out, such as flavonoids, alkaloids, unexplored proteins, and anti-tyrosinase compounds (Weng et al. 2003; Ko et al. 2004; Ukil et al. 2017; Cheng et al. 2020). In the previous study, the analysis based on its complete chloroplast genome is still lack. Here, to provide a solid foundation for further phylogenetic studies, we assembled and characterized the complete plastome of *C. pallida.*

In this study, the sample of *C. pallida* was collected from Dali, Yunnan province, China (100°15′42.75′′E, 25°31′33.38′′N). The voucher specimen (NO. ZSY109) was preserved at the Herbarium of Medicinal Plants and Crude Drugs of the College of Pharmacy, Dali University. We used the modified CTAB method (Doyle 1987; Yang et al. 2014) to extract the total genomic DNA from the dry and healthy leaves. A pair-end (2 × 300 bp) library was constructed and sequenced *via* the Illumina Hiseq 2500 (Novogene, Tianjing, China) platform. After the sequencing, the raw data were filtered *via* Trimmomatic version 0.32 with default settings (Bolger et al. 2014). Subsequently, the filtered sequences were assembled into circular contigs utilizing GetOrganelle (Jin et al. 2020) with the cp genome of closely related species *Lupinus atlanticus* (KU726827) as the reference. Finally, the assembled genome was annotated and corrected manually using Geneious R version 11.0.2 (Kearse et al. 2012). The annotated complete chloroplast genome of *C. pallida* was submitted to GenBank under the accession number of MT920364.

The complete chloroplast genomes of *C. pallida* were 152,658 bp in length. The typical quadripartite structure consisted of a pair of inverted repeat regions (IRa and IRb, 25,489 bp), a large single-copy region (LSC, 83,652 bp), and a small single-copy region (SSC, 18,028 bp). The total GC content was 36.7%, while those of IR regions (42.9%) were higher than LSC (34.3%) and SSC (30.6%) regions. A total of 111 genes was successfully annotated, containing 77 coding protein genes, 30 *tRNA* genes, and four *rRNA* genes.

To further ascertain the phylogenetic position of *C. pallida*, a phylogenetic analysis was carried out among 23 complete chloroplast genomes in Leguminosae with *Eriobotrya malipoensis* (Rosaceae) as an outgroup. The genome sequences were aligned with MAFFT version 7.427 (Katoh and Standley 2013) and then the maximum-likelihood (ML) tree was conducted by RAxML (Stamatakis 2014) program with 1000 bootstrap replicates and the GTRGAMMAI model. The result showed that *C. pallida* was closely related to *Lupinus* in the family of Leguminosae (Figure 1).

**Figure 1.**
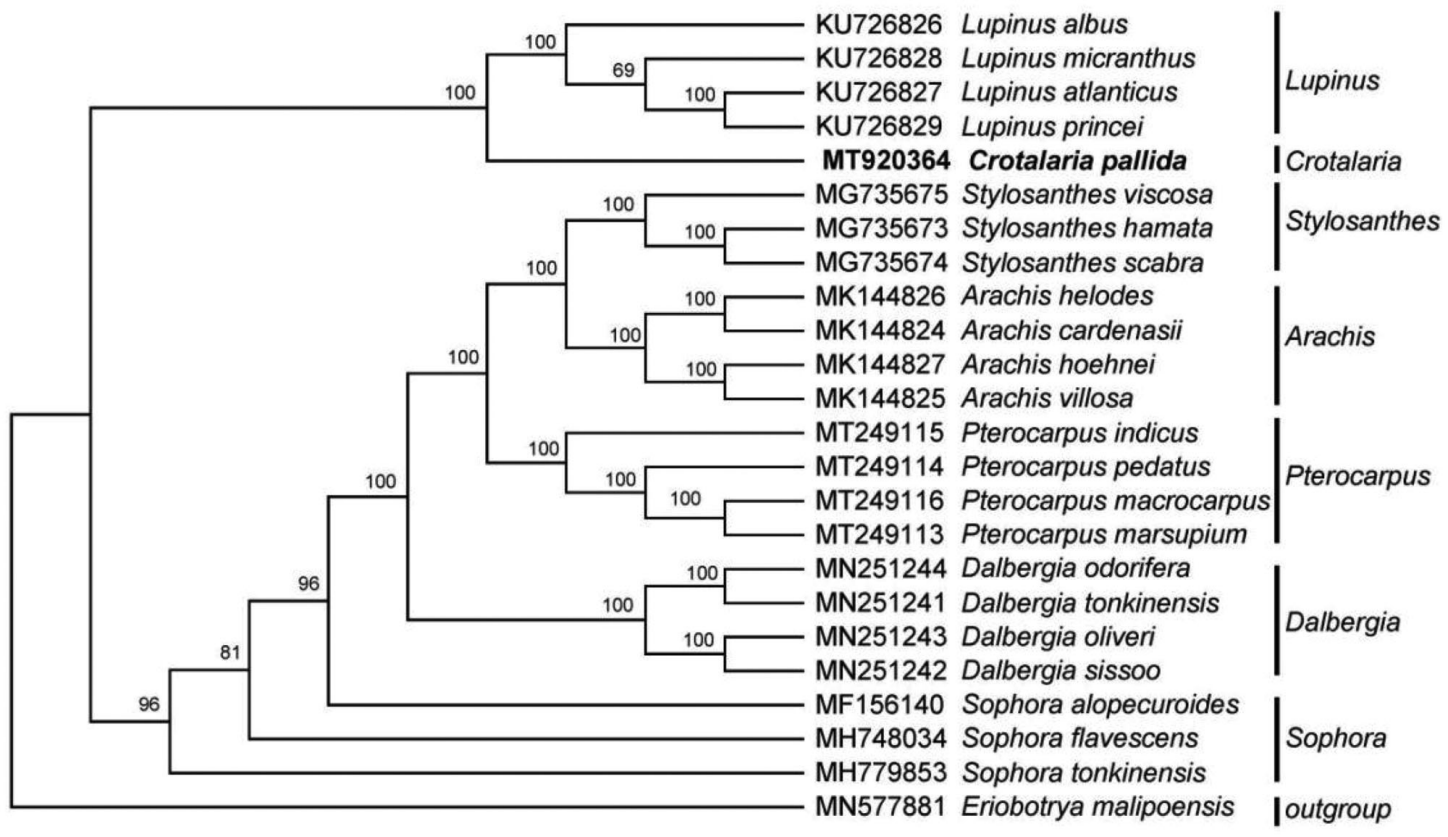
ML phylogenetic tree based on the complete chloroplast genome sequences of 24 species with *Eriobotrya malipoensis* as an outgroup. Bootstrap support values (1000 replicates) are shown next to the nodes.

## Data Availability

The data supporting the finding of this study is available in GenBank. The accession number is MT920364: https://www.ncbi.nlm.nih.gov/nuccore/MT920364.
